# Altered Regional Cerebral Blood Flow of Right Cerebellum Posterior Lobe in Asthmatic Patients With or Without Depressive Symptoms

**DOI:** 10.3389/fpsyt.2018.00225

**Published:** 2018-05-28

**Authors:** Yuqun Zhang, Yuan Yang, Ze Wang, Rongrong Bian, Wenhao Jiang, Yingying Yin, Yingying Yue, Zhenghua Hou, Yonggui Yuan

**Affiliations:** ^1^Department of Psychosomatics and Psychiatry, ZhongDa Hospital, School of Medicine, Southeast University, Nanjing, China; ^2^Institute of Psychosomatics, School of Medicine, Southeast University, Nanjing, China; ^3^Department of Respiration, ZhongDa Hospital, Southeast University, Nanjing, China; ^4^Center for Cognition and Brain Disorders and the Affiliated Hospital, Hangzhou Normal University, Hangzhou, China

**Keywords:** depression, asthma, cerebral blood flow, pulsed arterial spin labeling, cerebellum

## Abstract

**Background:** Asthma is a chronic disease appeared to be associated with depression. But the underpinnings of depression in asthma remain unknown. In order to understand the neural mechanisms of depression in asthma, we used cerebral blood flow (CBF) to probe the difference between depressed asthmatic (DA) and non-depressed asthmatic (NDA) patients.

**Methods:** Eighteen DA patients, 24 NDA patients and 57 healthy controls (HC) received pulsed arterial spin labeling (pASL) scan for measuring CBF, resting-state functional magnetic resonance imaging (rs-fMRI) scan, severity of depression and asthma control assessment, respectively.

**Results:** Compared to NDA, DA patients showed increased regional CBF (rCBF) in the right cerebellum posterior lobe. Compared to HC, DA, and NDA patients all showed significantly decreased rCBF in the right cerebellum posterior lobe.

**Conclusions:** We showed the first evidence of altered rCBF in the right cerebellum posterior lobe in asthma using pASL, which appeared to be involved in the neuropathology in asthma.

**Clinical Trial Registration:** An investigation of therapeutic mechanism in asthmatic patients: based on the results of Group Cognitive Behavioral Therapy (Registration number: ChiCTR-COC-15007442) (http://www.chictr.org.cn/usercenter.aspx).

## Introduction

Asthma was a chronic inflammatory condition that swelled and narrowed the airways, leading to dyspnea, coughing, and tightening of the chest. To et al. (1) showed the global prevalence rates of doctor-diagnosed asthma, clinical/treated asthma and wheezing in adults were 4.3, 4.5, and 8.6% respectively, and varied by as much as 21-fold amongst the 70 countries. Ding et al. (2) assessed the population of asthma in urban China with data from 2010 to 2013 in China National Health and Wellness Survey and reported that the prevalence of asthma was 30.73%. Several epidemiology studies consistently documented that depression was prevalent in patients with asthma, and was associated with uncontrolled asthma and poor quality of life (3–5).

Over the past decades, functional magnetic response imaging (fMRI) proved itself a useful technique to detect and quantitate sites of activation in the brain and to map circuits that might be associated with or involved in the underpinnings of emotion in asthma. Rosenkranz et al. (6–8) explored the neural circuitry underlying the interaction between emotion and asthma symptoms used task fMRI, the findings consistently indicated that neurophenotypes of asthma might be identified by neural activity of brain circuits previously implicated in emotion regulation, especially the insula and anterior cingulate cortex (ACC). In addition, dyspnea shared emotion-related brain network also has been suggested, including the insula, ACC, amygdala and medial thalamus (9). On the basis of previous studies, Busse (10) summarized minutely how was the central nervous system involved in allergic airway response in asthma and how this related to stress. This review emphasized the important role of emotion-related neural networks in asthma attack and maintenance processing. Subsequently, neural circuits turned to be a critical joint linked asthma and emotion. However, previous research predominantly focused on the neurobiological mechanisms of fear and anxiety in asthma, a better understanding of the biological pathogenesis of depression was required.

Increasing evidence revealed abnormal glucose metabolism and cerebral blood flow (CBF) of certain brain regions in depressed patients, such as hippocampus (11), right prefrontal and (12, 13) and striatal regions (12). In patients with chronic obstructive pulmonary disease (COPD), Yildiz et al. (14) reported increased CBF at rest because cerebral autoregulation-mediated vasodilatation to overcome COPD exacerbation induced hypoxia. Enhanced CBF appeared when healthy subjects experiencing hypoxia and hypercapnia (15), which might be explained by the tendency of upregulating PaCO_2_, a potent cerebral vasodilator (16). Considering abnormal ventilation of asthmatic patients, the aberrant PaCO_2_ level might also exist in certain brain regions. Unfortunately, studies exploring CBF in depressed asthmatic (DA) patients were not found, although the abnormalities were identified in both depression (11) and asthmatic (17) patients separately. Here, we adopted a method of arterial spin labeling (ASL) perfusion magnetic resonance imaging (MRI) which is a technique for quantifying regional brain perfusion and requiring no radioactive source or contrast agent (18), to investigate the regional CBF (rCBF) in asthmatic patients. ASL perfusion MRI renders the interpretation of quantitative measurements of the rCBF, a physiological parameter, easier than the assessment of the blood-oxygen-level-dependent (BOLD) effect at resting state (13, 19). Pulsed ASL (pASL) uses a short radio frequency pulse to invert the blood water spins in a very short time and provides signal-to-noise ratio as well as increased physiological noise (20).

To the current study's best knowledge, the pattern of rCBF changes in DA patients has not been characterized by pASL studies. So, a data-driven analysis was chosen to study the data according to the complexity and multidimensional causes of DA together with variability between individuals. We identified regions of interest (ROI) with abnormal rCBF between DA and non-depressed asthmatic (NDA), healthy controls (HC) groups. ROIs were then used as seeds to find out the rCBF alterations in DA patients and provided evidence for exploring the mechanism of depression in asthma.

## Materials and methods

### Participants and evaluations

**Figure 1:**
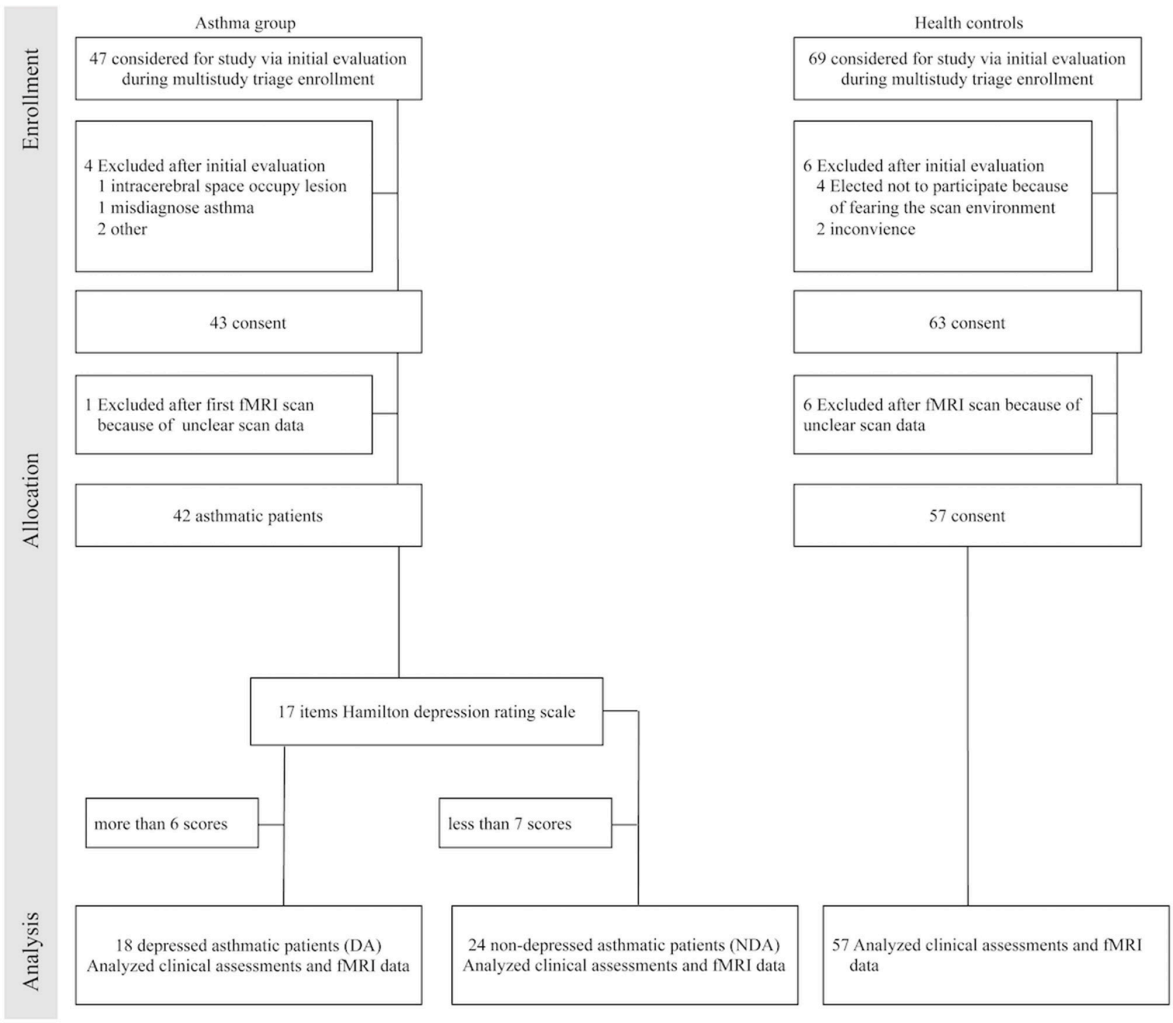
Flow diagram. fMRI indicated functional magnetic resonance imaging.

According to the scores of 17 items Hamilton depression rating scale (HDRS-17), asthmatic patients were divided into DA (HDRS-17 ≥ 7) and NDA (HDRS-17 < 7) group. As shown in Figure 1 (a flow diagram) and Table 1 (listing demographic data), 18 DA patients (mean age was 53.61 years, 9 males and 9 females), 24 NDA patients (mean age was 50.58 years, 9 males and 15 females) and 57 HC (mean age was 45.63 years, 23 males and 34 females) participated in this study after attrition and data cleaning. HDRS-17 was used to assess the depression of all participants. The asthma control test (ACT) is a self-rating scale and used to measure asthma control level in asthmatic patients. This study was carried out in accordance with the recommendations of the ethics committee of Zhongda Hospital with written informed consent from all subjects. All subjects gave written informed consent in accordance with the Declaration of Helsinki. The protocol was approved by the ethics committee of Zhongda Hospital, Southeast University. The clinical trial registration number was ChiCTR-COC-15007442.

**Table 1:** Demographics and clinical characteristics of participants.

	**DA (*n* = 18)**	**NDA (*n* = 24)**	**HC (*n* = 57)**	***P-value***
Age (years)	53.61 ± 9.08^*^	50.58 ± 10.57	45.63 ± 14.70	0.05^a^
Gender (male/female)	9/9	9/15	23/34	0.723^b^
Education (years)	11.89 ± 2.56	11.75 ± 2.64	12.42 ± 3.60	0.643^a^
Duration of asthma (years)	22.86 ± 20.19	21.41 ± 19.27	–	0.815^c^
HDRS-17 scores	11.06 ± 4.40^**^^††^	2.21 ± 1.47^#^	0.89 ± 1.35	<0.001^a^
ACT scores	15.00 ± 4.38	19.58 ± 4.31	–	0.002^c^

*Data are expressed as mean ± standard deviation*.

a One-way ANOVA;

b Chi-square test;

c *Independent-sample t-test*.

*DA vs. HC*,

* *P < 0.05*,

** *P < 0.001; DA vs. NDA*,

†† *P < 0.001; NDA vs. HC*,

# *P < 0.05. DA, depressed asthma; NDA, non-depressed asthma; HC, healthy controls; HDRS-17, 17 items Hamilton Depression Rating Scale; ACT, asthma control test*.

### Inclusion/exclusion criteria

Participants who met the following criteria entered asthma group: (1) met the diagnostic criteria of bronchial asthma and during non-acute attacks; (2) 18 years old or above; (3) right handedness; (4) education to junior high school or above; (5) there is no electronic and metal equipment in body (such as cardiac pacemaker, defibrillator, stent, et al.); (6) participants sign the informed consent form. Inclusion criteria of HC: HDRS-17 < 7 and the above item (2) ~ (6) in the inclusion criteria of asthmatic patients.

Participants with one of following items were excluded: (1) other serious disease of the respiratory system; (2) history of other mental disorders, alcohol, and drug dependence; (3) organic brain disorders and cardio, hepar, kidney abnormality; (5) women during pregnancy or lactation.

### Brain image acquisition

Imaging was performed on a 3-Tesla Siemens Magnetom Symphony scanner using a homogeneous birdcage head coil. Subjects laid supine with the head snugly fixed by a belt and foam pads to minimize head motion. High-resolution 3-dimensional T1-weighted scans were performed using the Siemens product magnetization prepared rapid gradient echo (MPRAGE) sequence [repetition time (TR) = 1,900 ms, echo time (TE) = 2.48 ms; flip angle (FA) = 9°; acquisition matrix = 256 × 256; field of view (FOV) = 250 × 250 mm^2^; thickness = 1.0 mm, gap = 0; time = 4 min 18 s]. ASL perfusion MRI was performed using the Siemens product pASL PICORE Q2T sequence (TR = 4,000 ms, TE = 12 ms; TI1 = 600 ms, TI2 = 1,600 ms; FA = 90°; maxtrix = 64 × 64; FOV = 220 × 220 mm^2^; 27 axial slices; thickness = 4 mm; gap = 1 mm, total scan time = 7 min 14 s).

### Preprocessing protocol

All the image data were reconstructed and inspected by two experienced radiologists. T1 images were manually checked for quality controls. pASL data were processed using SPM12 (http://www.fil.ion.ucl.ac.uk/spm) and ASLtbx (21). To avoid the spurious motion artifacts due to the systematic labeling and non-labeling in ASL, motion corrections were performed using the amended motion correction algorithm implemented in ASLtbx (21, 22). The raw ASL images were then high pass filtered to keep the higher half frequency band. The pASL images were then co-registered to the T1 images and spatially smoothed with a 6 mm full-width-half-maximum (FWHM) kernel, followed by pairwise control/label image subtraction and CBF quantification. After rejecting the outlier CBF volumes using the prior-guided adaptive outlier cleaning algorithm, mean CBF map was created from the remaining CBF volumes and were registered into the MNI space using the transformation obtained through the structural image.

### Statistical analysis

Predictive Analytic Software (PASW) Statistics 18 package was employed (IBM Corporation, Armonk, NY, USA) to complete the analyses. Age, education, and HDRS-17 scores were performed by one-way analysis of variance (ANOVA). Gender was compared by means of the Chi-square test. Duration of illness and ACT scores were analyzed by independent sample *t*-test. *P* < 0.05 were considered to indicate statistical significance.

CBF comparisons were processed with REST software (23). Statistical tests across groups were performed using a voxel-based, one-way analysis of covariance (ANCOVA), with age, gender and education level as covariates, followed by *post-hoc* two-sample *t*-tests. AlphaSim correction based on Monte Carlo simulation algorithm was used to correct for multiple comparisons [single voxel *P* value = 0.025, FWHM = 6 mm, with 61 × 73 × 61 mm^3^ gray matter mask, which yielded a corrected threshold of *P* < 0.025, cluster size > 2025 mm^3^/75 voxels (http://afni.nimh.nih.gov/pub/dist/doc/manual/AlphaSim.pdf)]. The *post-hoc* two-sample *t*-tests were conducted within a mask showing significant differences obtained from the ANCOVA analysis, with AlphaSim corrections (single voxel *P*-value = 0.025, FWHM = 6 mm, which yielded a corrected threshold of *P* < 0.025, cluster size > 162 mm^3^/6 voxels).

Brain regions were selected as ROI only when they were exhibiting significant differences both between the DA and NDA groups and between DA and HC groups. Mean CBF values were extracted within each of these ROIs, then Pearson correlation coefficients were computed between the extracted CBF values within these ROIs and the clinical assessments of DA patients by PASW 18.0, and the significance level was set at *P* < 0.05 (two-tailed).

## Results

### Demographic and clinical data

Table 1 showed the demographic and clinical variables. No significant differences in participants' gender, education, and durations of asthma were found between groups. The age of DA was significantly elder than HC (*P* < 0.05). It would be a covariate in the following statistical analysis of CBF. There was a significant difference in HDRS-17 scores among the three groups (*P* < 0.001). And the ACT scores were significantly different between DA and NDA group (*P* < 0.01).

### Group differences of CBF

**Figure 2:**
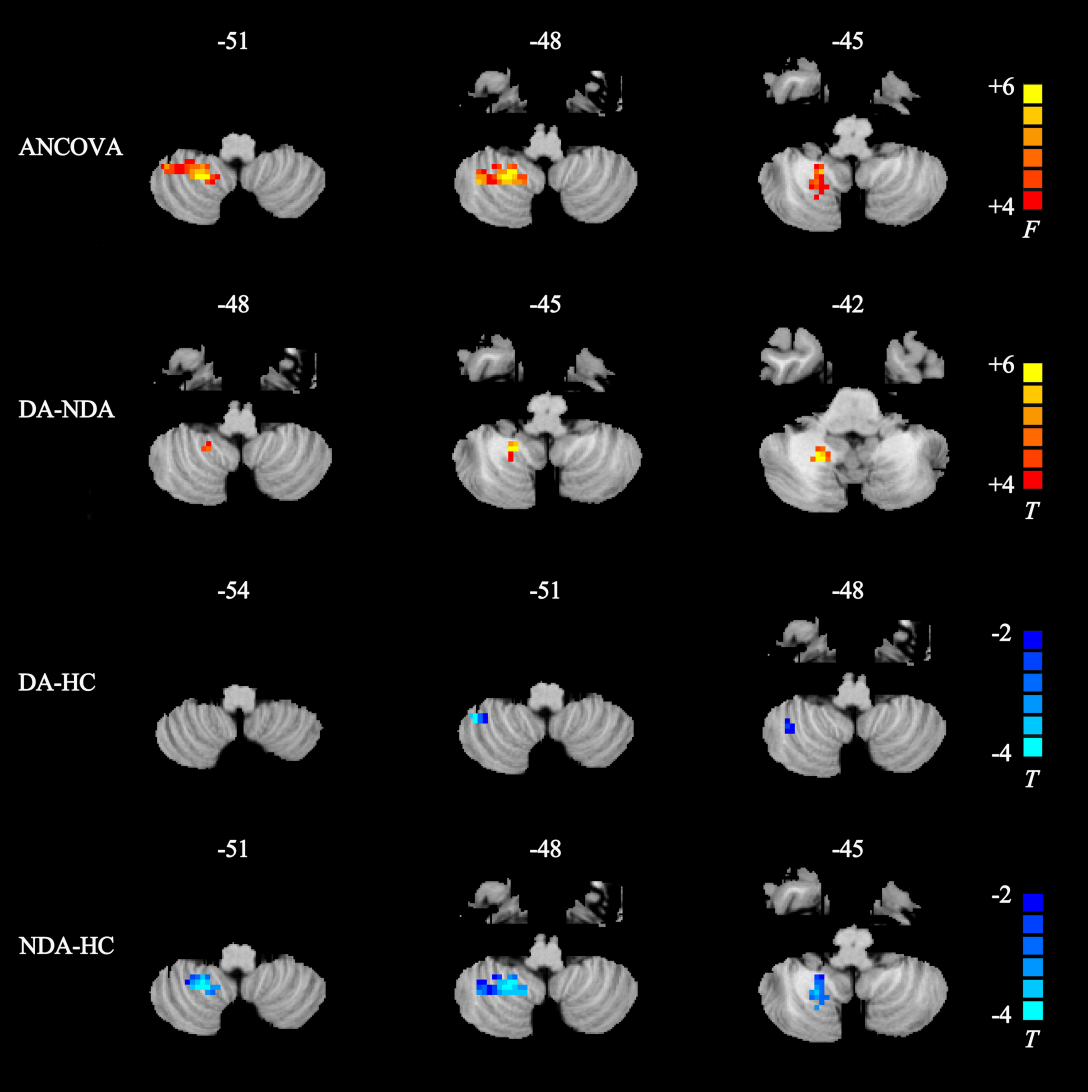
Statistical maps showing rCBF differences in different brain regions between DA, NDA, and HC groups. ANCOVA, significantly increased in rCBF among DA, NDA and HC groups (*P* < 0.025, AlphaSim corrected); Higher rCBF was in the right cerebellum posterior lobe; the red color bar indicates the *F-*value from ANCOVA among three groups. DA-NDA, significantly increased in rCBF of DA patients compared with NDA patients (*P* < 0.025, AlphaSim corrected); the DA patients showed higher CBF in the right cerebellum posterior lobe; the red color bar indicates the t value from independent sample *t*-test between DA and NDA groups. DA-HC, significantly decreased in rCBF of DA patients compared with HC (*P* < 0.025, AlphaSim corrected); the DA patients showed lower rCBF in the right cerebellum posterior lobe; the blue color bar indicates the t value from independent sample *t*-test between DA and HC groups. NDA-HC, significantly decreased in rCBF of NDA patients compared with HC patients (*P* < 0.025, AlphaSim corrected); the NDA patients showed lower rCBF in the right cerebellum posterior lobe; the blue color bar indicates the t value from independent sample *t*-test between NDA and HC groups. ANCOVA, analysis of covariance; DA, depressed asthma; NDA, non-depressed asthma; HC, healthy controls.

As displayed in Table 2 and Figure 2, the DA patients showed increased rCBF in the right cerebellum posterior lobe (CPL) compared than in NDA patients. In addition, DA patients exhibited lower rCBF in the right CPL compared with HC. Significantly decreased CBF value was also observed in the right CPL in the NDA group relative to HC.

**Table 2:** Regions showing significant differences in rCBF between groups.

**Peak area**	**Side**	**MNI coordinates**			**Voxels**	**Peak *t-*value**
		**X**	**Y**	**Z**		
**ANCOVA**						
Cerebellum posterior lobe	R	21	−51	−48	90	6.5089
**DA-NDA**						
Cerebellum posterior lobe	R	18	−51	−45	20	3.5013
**DA-HC**						
Cerebellum posterior lobe	R	42	−48	−51	12	−3.6889
**NDA-HC**						
Cerebellum posterior lobe	R	21	−51	−48	78	−3.4643

*ANCOVA, threshold was set at P < 0.025 (AlphaSim-corrected, cluster size > 2,025 mm^3^ voxels); Two-sample t-test, threshold was also set at P < 0.025 (AlphaSim-corrected, cluster size > 162 mm^3^ voxels). X, Y, Z, coordinates of primary peak locations in the MNI space. rCBF, regional cerebral blood flow; MNI, Montreal Neurological Institute space; BA, Brodmann area; R, right; ANCOVA, analysis of covariance; DA, depressed asthma; NDA, non-depressed asthma; HC, healthy controls*.

### Correlations between CBF values and clinical assessments

The present study used partial correlation analysis to explore the relationships between mean CBF values in the right CPL and two scales (HDRS-17 and ACT). It revealed that no significant correlations were found between CBF values and HDRS-17, ACT scores respectively either in DA or NDA group.

## Discussions

To our knowledge, this study at the first time used pASL method to explore the relationship between altered rCBF and depression in asthmatic patients. DA patients exhibited increased CBF values in the right CPL compared with NDA patients, and reversed result compared with HC.

Stoodley and Schmahmann (24) highlighted the widely functions of cerebellum, which included sensorimotor control, language, spatial, and executive functions. Moreover, positron emission tomography (PET) and fMRI studies have demonstrated that cerebellar activation was also involved in the emotional processing paradigms (24, 25), especially the right CPL (26, 27). It further supported our finding that DA patients showed increased rCBF in the right CPL compared with NDA. In patients with late-onset depression, the excessive cerebellar FC with medial prefrontal lobe displayed significant correlation with depression symptoms (28). Su et al. (29) summarized the cerebral metabolism of depression patients based on PET, suggesting that altered metabolism in cerebellum is likely to play a key role in the pathophysiology of depression. Several metabolism-related investigations suggested that the MDD patients exhibited enhanced metabolism in the right CPL (29–31). The previous study also reported decreased CBF in the cerebellum in patients with depression disorder (32), however the similar finding was not found in DA patients. Thus, the increased rCBF in the right CPL might be associated with the depression in asthmatic patients. However, in the current study, DA and NDA patients displayed decreased rCBF in the right CPL compared with HCs. We deduced that it might be influenced by other factors such as cognitive function (24).

Stoodley et al. (24, 33–36) made a series of studies to explore the function of human cerebellum through neuroimaging. Their findings demonstrated that the posterior lobe was also involved in higher-level tasks, including language and verbal working memory, spatial tasks, executive function besides emotional processing, especially the region of lobule VI which was reported in our study. For asthmatic patients, they followed doctors' recommendations less frequently, subsequently led to a vicious circle (37). However, underlying this were serious cognitive dysfunctions which contributed to difficulties in understanding given advice and putting it into practice (37, 38). In contrast, Ray et al. (39) reported that poor asthma control and airway obstruction were not associated with poor performance on various measures of cognitive function in older adults with asthma. Although limited research presented inconsistent results, cognitive dysfunction existed in asthmatic patients was consistent approbation. Unfortunately, we did not explore the relationship between asthma and cognitive function in the current study, research focusing on the related issues needed to be investigated in the future.

Evidence of aberrant activities in ACC and insula were often discovered whatever in emotion disorders or asthma (7–9, 40, 41). von Leupoldt et al. (9, 40, 42) explored a series studies of dyspnea in asthmatic patients and healthy participants used task fMRI. Their findings suggested that emotion-related brain regions including ACC and insula showed abnormal BOLD signal while experiencing dyspnea from mild to severe. However, both regions did not exhibit dysfunctions used the method of pASL in the current study. We detected that methodological differences (task vs. resting state and BOLD vs. pASL) would be the main possible reasons for the lack of ACC and insula dysfunctions in our study.

Significantly correlations between CBF values of the right CPL and HDRS-17 scores were not found whatever in DA or NDA group in the current study. A regional cerebral metabolism study suggested that different depressive symptom clusters might have different neural structures in unipolar depression, and depression symptom clusters predominantly correlated with the cerebral metabolism in right insula, temporal cortex, and ACC (43). CPL was a critical node possessed various functions (24), the insignificant correlation between the CBF values and HDRS-17 scores might be influenced by other confused factors (e.g., aberrant ventilation, cognitive function). It suggested that the altered rCBF in the right CPL might independent of depression severity and asthma control.

There are some limitations to our study. First, since the current study mainly focused on the differences of rCBF between asthmatic patients with and without depression, we adopted only HDRS-17 and ACT for the evaluation of depressive- and asthma control- level in participants. More cognitive-related tests were required to adequately describe patients cognitive profile, and to confirmed the speculations that the abnormal rCBF in right CPL might reflect impaired cognitive function in asthmatic patients. Second, our study involved a relatively small sample, and the number of the subjects in the three groups did not match perfectly. To control for the effects of the differences in age among the three groups, this variable was considered as covariates and regressed out in the statistical analysis.

In summary, this was the first study to explore the potential mechanism of depression in asthma using pASL. The findings demonstrated that the increased rCBF in the right CPL would be involved in the neuropathology of depression in asthma.

## Author contributions

YZ took responsibility for the content of the manuscript; YZ and ZW were responsibility for the data and analysis; YZ, YYang and RB were charge for patient recruitment; WJ, YYin, YYue, ZH provided language help for this study; YYuan was responsibility for experimental design.

### Conflict of interest statement

The authors declare that the research was conducted in the absence of any commercial or financial relationships that could be construed as a potential conflict of interest.

## References

[1] To T Stanojevic S Moores G Gershon AS Bateman ED Cruz AA. Global asthma prevalence in adults: findings from the cross-sectional world health survey. BMC Public Health (2012) 12:204. 10.1186/1471-2458-12-204 22429515 PMC3353191

[2] Ding B DiBonaventura M Karlsson N Ling X. Asthma-chronic obstructive pulmonary disease overlap syndrome in the urban Chinese population: prevalence and disease burden using the 2010, 2012, and 2013 China National Health and Wellness Surveys. Int J Chron Obstruct Pulmon Dis. (2016) 11:1139–50. 10.2147/COPD.S103873 27354777 PMC4907484

[3] Liu S Wu R Li L Liu L Li G Zhang X. The prevalence of anxiety and depression in Chinese asthma patients. PLoS ONE (2014) 9:e103014. 10.1371/journal.pone.0103014 25054657 PMC4108371

[4] Lu Y Ho R Lim TK Kuan WS Goh DY Mahadevan M. Psychiatric comorbidities in Asian adolescent asthma patients and the contributions of neuroticism and perceived stress. J Adolesc Health (2014) 55:267–75. 10.1016/j.jadohealth.2014.01.007 24630495

[5] Ciprandi G Schiavetti I Rindone E Ricciardolo FL. The impact of anxiety and depression on outpatients with asthma. Ann Allergy Asthma Immunol. (2015) 115:408–14. 10.1016/j.anai.2015.08.007 26392047

[6] Rosenkranz MA Busse WW Johnstone T Swenson CA Crisafi GM Jackson MM. Neural circuitry underlying the interaction between emotion and asthma symptom exacerbation. Proc Natl Acad Sci USA. (2005) 102:13319–24. 10.1073/pnas.0504365102 16141324 PMC1197272

[7] Rosenkranz MA Busse WW Sheridan JF Crisafi GM Davidson RJ. Are there neurophenotypes for asthma? Functional brain imaging of the interaction between emotion and inflammation in asthma. PLoS ONE (2012) 7:e40921. 10.1371/journal.pone.0040921 22870208 PMC3411610

[8] Rosenkranz MA Davidson RJ. Affective neural circuitry and mind-body influences in asthma. Neuroimage (2009) 47:972–80. 10.1016/j.neuroimage.2009.05.042 19465136 PMC2748325

[9] von Leupoldt A Sommer T Kegat S Baumann HJ Klose H Dahme B. Dyspnea and pain share emotion-related brain network. Neuroimage (2009) 48:200–6. 10.1016/j.neuroimage.2009.06.015 19527787

[10] Busse WW. The brain and asthma: what are the linkages? Chem Immunol Allergy (2012) 98:14–31. 10.1159/000336495 22767055

[11] Suzuki H Matsumoto Y Ota H Sugimura K Takahashi J Ito K. Hippocampal blood flow abnormality associated with depressive symptoms and cognitive impairment in patients with chronic heart failure. Circ J (2016) 80:1773–80. 10.1253/circj.CJ-16-0367 27295999

[12] Cantisani A Koenig T Stegmayer K Federspiel A Horn H Müller TJ. EEG marker of inhibitory brain activity correlates with resting-state cerebral blood flow in the reward system in major depression. Eur Arch Psychiatry Clin Neurosci. (2015) 266:755–64. 10.1007/s00406-015-0652-7 26590845

[13] Kaichi Y Okada G Takamura M Toki S Akiyama Y Higaki T. Changes in the regional cerebral blood flow detected by arterial spin labeling after 6-week escitalopram treatment for major depressive disorder. J Affect Disord. (2016) 194:135–43. 10.1016/j.jad.2015.12.062 26826533

[14] Yildiz S Kaya I Cece H Gencer M Ziylan Z Yalcin F. Impact of COPD exacerbation on cerebral blood flow. Clin Imaging (2012) 36:185–90. 10.1016/j.clinimag.2011.08.021 22542376

[15] Curran-Everett D Zhang Y Jones RH Jones MD Jr. Hypoxia, hypercapnia, and hypertension: their effects on pulsatile cerebral blood flow. J Appl Physiol. (1995). 79:870–8. 10.1152/jappl.1995.79.3.870 8567530

[16] Ogoh S Ainslie PN. Cerebral blood flow during exercise: mechanisms of regulation. J Appl Physiol. (2009) 107:1370–80. 10.1152/japplphysiol.00573.2009 19729591

[17] Bowton DL Stump DA Anderson R. Effect of chronic theophylline therapy on brain blood flow and function in adult asthmatics. Am J Respir Crit Care Med. (1994) 150:1002–5. 10.1164/ajrccm.150.4.7921428 7921428

[18] Detre JA Alsop DC. Perfusion magnetic resonance imaging with continuous arterial spin labeling: methods and clinical applications in the central nervous system. Eur J Radiol. (1999) 30:115–24. 10.1016/S0720-048X(99)00050-9 10401592

[19] Zhu S Fang Z Hu S Wang Z Rao H. Resting state brain function analysis using concurrent BOLD in ASL perfusion fMRI. PLoS ONE (2013) 8:e65884. 10.1371/journal.pone.0065884 23750275 PMC3672100

[20] Wang Z. Characterizing early Alzheimer's disease and disease progression using hippocampal volume and arterial spin labeling perfusion MRI. J Alzheimers Dis. (2014) 42(Suppl. 4):S495–502. 10.3233/JAD-141419 25182742

[21] Wang Z Aguirre GK Rao H Wang J Fernández-Seara MA Childress AR. Empirical optimization of ASL data analysis using an ASL data processing toolbox: ASLtbx. Magn Reson Imaging (2008) 26:261–9. 10.1016/j.mri.2007.07.003 17826940 PMC2268990

[22] Wang Z. Improving cerebral blood flow quantification for arterial spin labeled perfusion MRI by removing residual motion artifacts and global signal fluctuations. Magn Reson Imaging (2012) 30:1409–15. 10.1016/j.mri.2012.05.004 22789842 PMC3482282

[23] Song XW Dong ZY Long XY Li SF Zuo XN Zhu CZ. REST: a toolkit for resting-state functional magnetic resonance imaging data processing. PLoS ONE (2011) 6:e25031. 10.1371/journal.pone.0025031 21949842 PMC3176805

[24] Stoodley CJ Schmahmann JD. Functional topography in the human cerebellum: a meta-analysis of neuroimaging studies. Neuroimage (2009) 44:489–501. 10.1016/j.neuroimage.2008.08.039 18835452

[25] Van Overwalle F Baetens K Mariën P Vandekerckhove M. Social cognition and the cerebellum: a meta-analysis of over 350 fMRI studies. Neuroimage (2014) 86:554–72. 10.1016/j.neuroimage.2013.09.033 24076206

[26] Paradiso S Robinson RG Boles Ponto LL Watkins GL Hichwa RD. Regional cerebral blood flow changes during visually induced subjective sadness in healthy elderly persons. J Neuropsychiatry Clin Neurosci. (2003) 15:35–44. 10.1176/jnp.15.1.35 12556569

[27] Wildgruber D Riecker A Hertrich I Erb M Grodd W Ethofer T. Identification of emotional intonation evaluated by fMRI. Neuroimage (2005) 24:1233–41. 10.1016/j.neuroimage.2004.10.034 15670701

[28] Yin Y Hou Z Wang X Sui Y Yuan Y. Association between altered resting-state cortico-cerebellar functional connectivity networks and mood/cognition dysfunction in late-onset depression. J Neural Transm. (2015) 122:887–96. 10.1007/s00702-014-1347-3 25466433

[29] Su L Cai Y Xu Y Dutt A Shi S Bramon E. Cerebral metabolism in major depressive disorder: a voxel-based meta-analysis of positron emission tomography studies. BMC Psychiatry (2014) 14:321. 10.1186/s12888-014-0321-9 25407081 PMC4240898

[30] Hwang JP Lee TW Tsai SJ Chen TJ Yang CH Lirng JF. Cortical and subcortical abnormalities in late-onset depression with history of suicide attempts investigated with MRI and voxel-based morphometry. J Geriatr Psychiatry Neurol. (2010) 23:171–84. 10.1177/0891988710363713 20430976

[31] Fujimoto T Takeuchi K Matsumoto T Fujita S Honda K Higashi Y. Metabolic changes in the brain of patients with late-onset major depression. Psychiatry Res. (2008) 164:48–57. 10.1016/j.pscychresns.2007.03.010 18804352

[32] Liao W Wang Z Zhang X Shu H Wang Z Liu D. Cerebral blood flow changes in remitted early- and late-onset depression patients. Oncotarget (2017) 8:76214–22. 10.18632/oncotarget.19185 29100305 PMC5652699

[33] Stoodley CJ Schmahmann JD. Evidence for topographic organization in the cerebellum of motor control versus cognitive and affective processing. Cortex (2010) 46:831–44. 10.1016/j.cortex.2009.11.008 20152963 PMC2873095

[34] Stoodley CJ. The cerebellum and cognition: evidence from functional imaging studies. Cerebellum (2012) 11:352–65. 10.1007/s12311-011-0260-7 21373864

[35] Stoodley CJ Valera EM Schmahmann JD. An fMRI study of intra-individual functional topography in the human cerebellum. Behav Neurol. (2010) 23:65–79. 10.1155/2010/840942 20714062 PMC3776583

[36] Stoodley CJ Valera EM Schmahmann JD. Functional topography of the cerebellum for motor and cognitive tasks: an fMRI study. Neuroimage (2012) 59:1560–70. 10.1016/j.neuroimage.2011.08.065 21907811 PMC3230671

[37] Bratek A Zawada K Beil-Gawełczyk J Beil S Sozanska E Krysta K. Depressiveness, symptoms of anxiety and cognitive dysfunctions in patients with asthma and chronic obstructive pulmonary disease (COPD): possible associations with inflammation markers: a pilot study. J Neural Transm. (2015) 122(Suppl. 1):S83–91. 10.1007/s00702-014-1171-9 24532256 PMC4529448

[38] O'Conor R Wolf MS Smith SG Martynenko M Vicencio DP Sano M. Health literacy, cognitive function, proper use, and adherence to inhaled asthma controller medications among older adults with asthma. Chest (2015) 147:1307–15. 10.1378/chest.14-0914 25275432 PMC4420182

[39] Ray M Sano M Wisnivesky JP Wolf MS Federman AD. Asthma control and cognitive function in a cohort of elderly adults. J Am Geriatr Soc. (2015) 63:684–91. 10.1111/jgs.13350 25854286 PMC4406794

[40] von Leupoldt A Sommer T Kegat S Eippert F Baumann HJ Klose H. Down-regulation of insular cortex responses to dyspnea and pain in asthma. Am J Respir Crit Care Med. (2009) 180:232–8. 10.1164/rccm.200902-0300OC 19483110

[41] Herigstad M Hayen A Wiech K Pattinson KT. Dyspnoea and the brain. Respir Med. (2011) 105:809–17. 10.1016/j.rmed.2010.12.022 21295457

[42] von Leupoldt A Brassen S Baumann HJ Klose H Buchel C. Structural brain changes related to disease duration in patients with asthma. PLoS ONE (2011) 6:e23739. 10.1371/journal.pone.0023739 21886820 PMC3158798

[43] Dunn RT Kimbrell TA Ketter TA Frye MA Willis MW Luckenbaugh DA. Principal components of the Beck Depression Inventory and regional cerebral metabolism in unipolar and bipolar depression. Biol Psychiatry (2002) 51:387–99. 10.1016/S0006-3223(01)01244-6 11904133

